# Local Mast Cell Activation Promotes Neovascularization

**DOI:** 10.3390/cells9030701

**Published:** 2020-03-12

**Authors:** Ilze Bot, Daniël van der Velden, Merel Bouwman, Mara J. Kröner, Johan Kuiper, Paul H. A. Quax, Margreet R. de Vries

**Affiliations:** 1 Division of BioTherapeutics, Leiden Academic Centre for Drug Research, Leiden University, 2333CC Leiden, The Netherlands; i.bot@lacdr.leidenuniv.nl (I.B.); daniel.vandervelden@hu.nl (D.v.d.V.); merel_bouwman@hotmail.com (M.B.); marakroner@hotmail.com (M.J.K.); j.kuiper@lacdr.leidenuniv.nl (J.K.); 2 Department of Surgery, Leiden University Medical Center, 2300RC Leiden, The Netherlands; p.h.a.quax@lumc.nl; 3 Einthoven Laboratory for Experimental Vascular Medicine, Leiden University Medical Center, 2300RC Leiden, The Netherlands

**Keywords:** arteriogenesis, angiogenesis, innate immunity, mast cell

## Abstract

Mast cells have been associated with arteriogenesis and collateral formation. In advanced human atherosclerotic plaques, mast cells have been shown to colocalize with plaque neovessels, and mast cells have also been associated with tumor vascularization. Based on these associations, we hypothesize that mast cells promote angiogenesis during ischemia. In human ischemic muscle tissue from patients with end-stage peripheral artery disease, we observed activated mast cells, predominantly located around capillaries. Also, in mouse ischemic muscles, mast cells were detected during the revascularization process and interestingly, mast cell activation status was enhanced up to 10 days after ischemia induction. To determine whether mast cells contribute to both arteriogenesis and angiogenesis, mast cells were locally activated immediately upon hind limb ischemia in C57Bl/6 mice. At day 9, we observed a 3-fold increase in activated mast cell numbers in the inguinal lymph nodes. This was accompanied by an increase in the amount of Ly6C^high^ inflammatory monocytes. Interestingly, local mast cell activation increased blood flow through the hind limb (46% at day 9) compared to that in non-activated control mice. Histological analysis of the muscle tissue revealed that mast cell activation did not affect the number of collaterals, but increased the collateral diameter, as well as the number of CD31^+^ capillaries. Together, these data illustrate that locally activated mast cell contribute to arteriogenesis and angiogenesis.

## 1. Introduction

The mast cell, part of the innate immune system, generally resides in tissues such as the lung and the skin to protect against pathogens like bacteria and parasites. Mast cells have also been described to participate in diseases such as asthma, allergies, and rheumatoid arthritis. Over the last decades, the mast cell has been implicated in cardiovascular diseases as well, for example in atherosclerosis [1,2], the underlying pathology of acute cardiovascular diseases including peripheral artery disease (PAD) [3,4]. Interestingly, mast cell numbers in advanced human atherosclerotic plaques obtained after endarterectomy surgery were seen to be of predictive value for the incidence of a secondary cardiovascular event [5]. In those plaques, mast cell density associated with the number of CD31^+^ microvessels [5]. Mast cells have also been associated with arteriogenesis and collateral formation [6]. Mast cells can secrete growth factors and pro-inflammatory cytokines that can recruit immune cells such as neutrophils and monocytes to the site of inflammation, thus further enhancing a pro-inflammatory response [6,7,8]. Patients with systemic mastocytosis, a disease that is characterized by the excessive accumulation of mast cells in tissue or organs, experienced an increased prevalence of cardiovascular disease events, such as myocardial infarction, stroke, and importantly, peripheral artery disease, which is actually caused by the development of atherosclerosis in the arteries that supply oxygen to the extremities [9]. These associative data suggest that the mast cell may actively contribute to these underlying pathologies, and preclinical studies have been performed to elucidate underlying mast cell pathways that are causally related to vascular remodeling and the related disease outcome.

We speculate that mast cells may affect atherosclerotic plaque progression and PAD by increasing angiogenesis. Plaque angiogenesis has been shown to be related to atherosclerotic plaque progression and also, during tumor development, mast cells have been implicated in angiogenesis [10]. Mast cells have been shown to induce tumor endothelial proliferation by the release of Vascular Endothelial Growth Factor (VEGF) in response to the hypoxic environment in the tumor [10], which may be translatable to muscles in PAD patients, in which hypoxia occurs as well [11,12]. Although induction of neovascularization, in particular angiogenesis, maybe unfavorable for atherosclerosis progression, in PAD, the induction of neovascularization by mast cells, more precisely by inducing collateral formation and angiogenesis, may act as a repair pathway to resupply the ischemic limb tissue with oxygen. This neovascularization process requires a pro-inflammatory response, which is thus, on one hand, beneficial to resolve ischemia, but may on the other hand lead to enhanced progression of atherosclerosis. This two-faced process is also known as the Janus phenomenon [13]. Recently, the contribution of mast cells to arteriogenesis and collateral formation in a shear-stress induced mouse model of hind limb ischemia (HLI) has been described by Chillo and colleagues [6]. In that study, mast cells were systemically activated with the mast cell activator compound 48/80 upon ligation of the femoral artery, which resulted in increased hind limb perfusion. Treatment with the mast cell stabilizer cromolyn prevented the mast cell-induced effects on arteriogenesis and the investigators identified the neutrophil as a prominent effector cell involved in the mechanism behind mast cell-induced arteriogenesis. However, in that study, mast cell activation-dependent effects on angiogenesis in the ischemic muscles were not reported. 

In this study, we aimed to investigate whether local induction of mast cell activation can stimulate blood flow recovery in a mouse hind limb ischemia model by inducing angiogenesis as well as arteriogenesis. 

First, we analyzed the number and activation status of mast cells in human ischemic tissue, obtained after limb amputation. Next, we induced hind limb ischemia in mice by ligation of the femoral artery and at time of ligation, we activated mast cells in the hind limb by a skin sensitization/challenge protocol using a pluronic gel to apply the hapten DNP locally at the ligation site as described before [7,14], after which blood perfusion was measured using laser Doppler imaging. Mast cell activation increased reperfusion of the hind limb by not only increasing arteriogenesis, but also angiogenesis, which is at least partly induced by increasing the pro-inflammatory monocyte response.

## 2. Materials and Methods

### 2.1. Tissue Collection from Patients with End-Stage Peripheral Artery Disease

Sample collection of tissues from patients with end-stage peripheral artery disease undergoing limb amputation was approved by the Medical Ethics Committee of the Leiden University Medical Center (Protocol No. P12.265). Written informed consent was obtained from the participants. Inclusion criteria were a minimum age of 18 years and lower limb amputation, excluding ankle, foot, or toe amputations. Exclusion criteria were suspected or confirmed malignancy and inability to give informed consent. Gastrocnemius and soleus muscle samples obtained from 15 patients were formalin fixed, processed, paraffin embedded sectioned, and stained for mast cells. From these patients, *n* = 1 had type I diabetes and *n* = 7 suffered from type II diabetes.

### 2.2. Hind Limb Ischemia Model

This study was performed in accordance with the Directive 2010/63/EU of the European Parliament and Dutch government guidelines. All experiments were approved (reference number 14185) by the Leiden University and Leiden University Medical Center committee on animal welfare (Leiden, the Netherlands). Wild-type C57Bl/6J mice were bred in our in-house breeding facility. Male mice aged 8 to 12 weeks were housed in groups with free access to water and regular chow.

Before the unilateral hind limb ischemia, mice were anesthetized by i.p. injection of midazolam (8 mg/kg, Roche Diagnostics, Basel, Switzerland), medetomidine (0.4 mg/kg, Orion, Espoo, Finland), and fentanyl (0.08 mg/kg, Janssen Pharmaceuticals, Beerse, Belgium). Hind limb ischemia was induced by electrocoagulation on two locations of the left femoral artery; the first ligation proximal to the superficial epigastric artery and the second proximal to the bifurcation of the popliteal and saphenous artery [15,16]. After surgery, anesthesia was antagonized with with atipamezol (2.5 mg/kg, Orion, Espoo, Finland) and flumazenil (0.5 mg/kg, Fresenius Kabi, Bad Homburg vor der Höhe, Germany).and buprenorphine (0.1 mg/kg, MSD Animal Health, Keniworth, NJ, USA) was provided as a painkiller. For the time course, 5 mice per time point were used, whereas for both the long-term (t28) and short-term (t9) HLI experiments, 8–9 mice per group were used. 

### 2.3. Local Mast Cell Activation with DPN treatment

Mice were skin-sensitized on the shaved abdomen and paws for 2 consecutive days with a dinitrofluorobenzene (DNFB (D1529) solution (0.5% *v*/*v* in acetone:olive oil (4:1), Sigma-Aldrich, St. Louis, MO, USA) as described previously to sensitize the mice for the hapten DNP [7,14]. In the control mice, a vehicle solution of acetone:olive oil (4:1) was applied. At the end of the hind limb ischemia procedure, which was scheduled one week after the skin-sensitization procedure, 50 µg dinitrophenyl hapten (DNP (D198501), Sigma-Aldrich, St. Louis, MO, USA) in a pluronic gel (25% *w*/*v*, Sigma-Aldrich, St. Louis, MO, USA) was applied around the ligated areas of the left hind limb to locally activate the mast cells. Empty pluronic gel was applied in the control mice. This model has been previously applied [7,14,17,18] and has been shown to specifically induce mast cells activation upon local hapten application.

### 2.4. Laser Doppler Perfusion Measurements

Before and directly after surgery and at 3, 7,10, 14, 21, and 28 days after surgery, blood flow recovery to the ligated hind limb and the unligated control paw were measured using Laser Doppler Perfusion Imaging (LDPI) (Moor Instruments, Axminster, UK). Before the LDPI measurements, mice were anesthetized by i.p. injection of midazolam (8 mg/kg) and medetomidine (0.4 mg/kg,). Next, mice were placed in a double glazed pot, perfused with water at 37 °C for 5 min [19]. After LDPI, anesthesia was antagonized by subcutaneous injection of flumazenil (0.7 mg/kg). LDPI measurements in the ligated paw were normalized to measurements of the unligated paw, as an internal control. At sacrifice, after the last LDPI measurement, analgesic fentanyl (0.08 mg/kg) was administered, blood was drawn, and mice were sacrificed via cervical dislocation. The adductor and soleus muscles were harvested and fixed in 4% formaldehyde. For a second short experiment, animals were sacrificed at day 9, where blood was collected by orbital bleeding and the inguinal lymph nodes were isolated for further analyses. Again, the adductor and soleus muscles were harvested and fixed in 4% formaldehyde for histology analysis.

### 2.5. Immunohistochemistry

**Figure 1:**
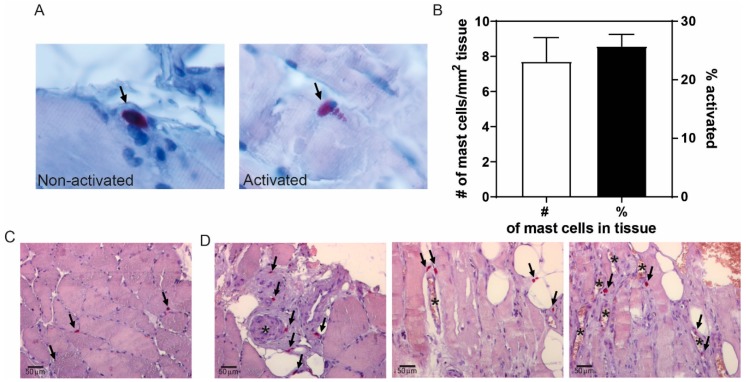
Mast cells in calf muscles of patients with peripheral artery disease. (**A**) Representative high-magnification image of a non-activated (left) and activated (right) mast cell, stained using a chloro-acetate esterase (CAE) staining and indicated by arrows. The non-activated mast cell shows the pink granula in the cytoplasm of the cell, whereas the pink granula are being released in the surroundings of the activated mast cell. (**B**) Quantification of the number of mast cells/mm^2^ tissue, and the percentage of activated mast cells observed (*n* = 15). (**C**) Overview of a chloro-acetate esterase (CAE) staining of muscle tissue showing mast cells in pink (indicate by arrows) in between muscle fibers. (**D**) Representative overview images of mast cells surrounding microvessels (indicated by *) in human calf muscle tissue.

Mast cells were visualized by staining using a naphthol AS-D chloroacetate esterase staining kit (#91C, Sigma-Aldrich, St. Louis, MO, USA) and counted manually. A mast cell was considered resting when all granula were maintained inside the cell, while mast cells were assessed as activated when granula were deposited in the tissue surrounding the mast cell (examples are shown in Figure 1A). Tissue size was quantified by the Leica image analysis system (Leica Ltd., Cambridge, UK). Paraffin embedded adductor and soleus muscle were stained with alpha smooth muscle actin (aSMA) (1A4, DAKO, Glostrup, Denmark) to visualize smooth muscle cell positive collaterals. Soleus muscles were stained for capillaries using CD31 (Sc-1506, Santa Cruz, Dallas, TX, USA) and macrophages using MAC3 (550292, BD-Pharmingen, Franklin Lakes, NJ, USA) together with aSMA and DAPI (Sigma, Santa Clara, CA, USA) as nuclear staining. For each antibody, negative controls were included using specific isotype-matched antibodies. The Pannoramic MIDI digital slide scanner (3DHistech, Budapest, Hungary) was used to create high-resolution images of the muscles. Snapshots (9 representative images per muscle) were taken using the caseviewer software (3DHistech) with a 40× magnification. aSMA positive collaterals were analyzed by counting the number of collaterals and measuring the diameters of each collateral with a visible lumen to determine arteriogenesis. CD31 positive capillaries and the number of MAC3 positive macrophages were quantified. All quantifications were performed using FIJI Image J image analysis software (ImageJ, Bethesda, MD, USA). 

### 2.6. FACS Analysis

Blood was collected at sacrifice, after which red blood cells were lysed using an erythrocyte lysis buffer (0.1 mM EDTA, 10 mM NaHCO_3_, 1 mM NH_4_Cl, pH = 7.2). Subsequently, white blood cells were stained with the antibodies for flow cytometric analysis. Inguinal lymph nodes were harvested from all mice and processed through a 70 μm cell strainer to acquire single cell suspensions. Subsequently, the cell suspensions were stained for flow cytometry. In approximation, 200,000 cells per sample were stained with antibodies against extracellular proteins at a concentration of 0.1 μg/sample for 30 min as described previously [20,21]. All flow cytometry experiments were executed on a FACS Canto II (BDBiosciences, San Jose, CA, USA) and data were analyzed using FlowJo software (v10, BDBiosciences).

### 2.7. Statistical Analysis

Results are presented as mean ± standard error of the mean (SEM). A 2-tailed Student’s t-test was used to compare individual groups. Non-Gaussian distributed data were analyzed using a 2-tailed Mann–Whitney U test. *p*-values < 0.05 were considered statistically significant and are indicated with *; *p*-values < 0.01 and < 0.001 are indicated by ** and ***, respectively. 

## 3. Results

### 3.1. Mast Cells Localization in Calf Muscles of Peripheral Artery Disease Patients

To assess mast cell presence and its activation status in calf muscles of peripheral artery disease patients, we stained sections of ischemic muscle biopsies obtained after amputation surgery with a CAE staining and quantified the number of mast cells. On average, we detected 7.7 ± 1.4 mast cells/mm^2^ tissue, of which 26 ± 2% was activated (Figure 1B). As shown in Figure 1C, mast cells, primarily non-activated, can be detected between the muscle fibers. Interestingly, mast cells, and in particular activated ones, generally appeared to colocalize with blood vessels in the muscle tissue (representative overview images in Figure 1D). The amount of mast cells in muscle tissue did not differ between patients that suffered from type I or II diabetes vs. patients that did not (7.5 ± 2.0 mast cells/mm^2^ vs. 7.4 ± 2.1 mast cells/mm^2^, respectively). Also, mast cell activation status in the muscle tissue was not affected by presence of diabetes (DM: 24.1 ± 2.3% vs. non-DM: 29.9 ± 3.1%). 

### 3.2. Activation of Mast Cells upon Ischemia in a Murine Hindlimb Ischemia Model

**Figure 2:**
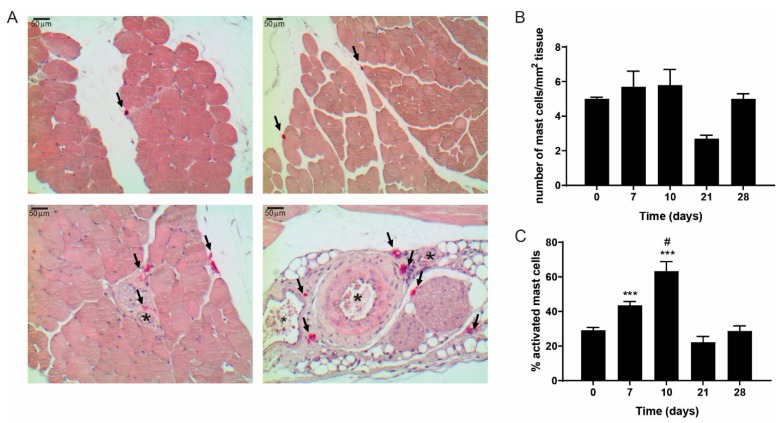
Mast cells in hind limb muscle tissue during ischemia in mice. (**A**) Representative images of mast cells in pink (CAE staining, indicated by arrows) in mouse ischemic muscle tissue (upper panels) and in close proximity to blood vessels (lower panels, indicated by *). (**B**) Mast cell density and (**C**) the percentage of activated mast cells in the hind limb muscle during recovery after ischemia. *p*-values of < 0.001 in comparison to t0 are indicated by *** *p*-value of < 0.05 in comparison to t7 is indicated by #.

To study whether mast cells accumulate in the murine muscle tissue upon hind limb ischemia, we quantified mast cell number and its activation status in adductor muscle tissue at 0, 7, 10, 21, and 28 days after the induction of hind limb ischemia. Similar to the human tissue, in murine muscles, mast cells were located in between muscle fibers and near blood vessels (Figure 2A). As the overall number of mast cells in this ischemic muscle tissue was relatively low, we included overview images of different locations in tissue to illustrate where mast cells reside (Figure 2A) Mast cell density, i.e., the number of mast cells per mm^2^ tissue, did not change during the time-course of the experiment (Figure 2B), but mast cell activation status increased upon the induction of ischemia, in particular during the initial phase from day 0 up to day 10 (Figure 2C).

At day 7, a 33% increase in activated mast cells was observed compared to the percentage of activated mast cells at day 0, which further rose to a maximum of a 54% increase at 10 days after HLI. The number of activated mast cells then declined at 21 and 28 days to the level control (day 0; non-surgery) muscles (Figure 2B). Interestingly, most of these activated mast cells, as indicated by granules that reside outside the cell, were found in close proximity of capillaries in the muscle tissues (Figure 2A, lower panels) suggesting a direct relation between blood vessels and mast cells as we have shown previously in atherosclerosis [5].

### 3.3. Effect of Local Mast Cell Activation on Post Ischemic Blood Flow Recovery.

**Figure 3:**
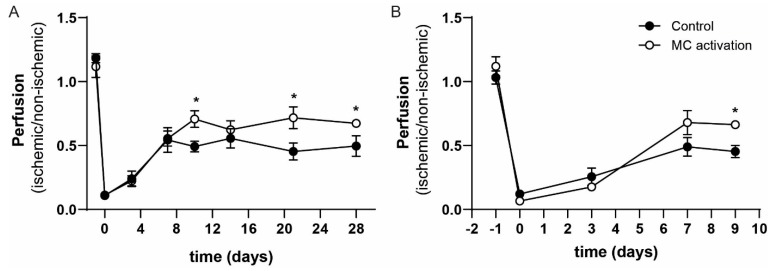
Perfusion of the ischemic hind limb in mast cell (MC) activated vs. control mice. Perfusion (ischemic/non-ischemic) as measured by Laser Doppler Perfusion Imaging (LDPI) from day 0 to day 28 in the long-term experiment (**A**) and in the short-term experiment from day 0 up to day 9 (**B**) after ligation of the femoral artery. * *p*-value of < 0.05 between the groups.

To study the effects of local mast cell activation on blood flow recovery, we applied the hapten DNP as part of our mast cell activation protocol on the gastrocnemius and adductor muscles directly after the hind limb ischemia procedure. We studied the subsequent blood flow recovery starting with eight mice per group, however between surgery and day 3, two mice in the DNP group and three mice in the control group had to be sacrificed because they reached humane end points. At day 10, a significant 44% increase in paw perfusion could be observed in the DNP treated group (left/right ratio of 0.70 ± 0.07) in comparison to the vehicle treated control group (left/right ratio of 0.49 ± 0.04). The enhanced increase in blood flow perfusion continued until the end of the experiment with significant differences between the groups at day 21 and day 28 (Figure 3A). We repeated the experiment, focusing on the early stage of blood flow recovery by sacrificing the mice at day 9 (short-term experiment). The experiment was started with nine mice per group; one mouse in the control and two mice in the DNP group reached the humane end point and were not included in the analysis. Similar to the t28 experiment, we observed an increase in paw perfusion in the DNP group at the day 9 time point of 46% (DNP: 0.66 ± 0.02 vs. control: 0.45 ± 0.05, Figure 3B).

### 3.4. Local Mast Cell Activation Induced by DNP 

**Figure 4:**
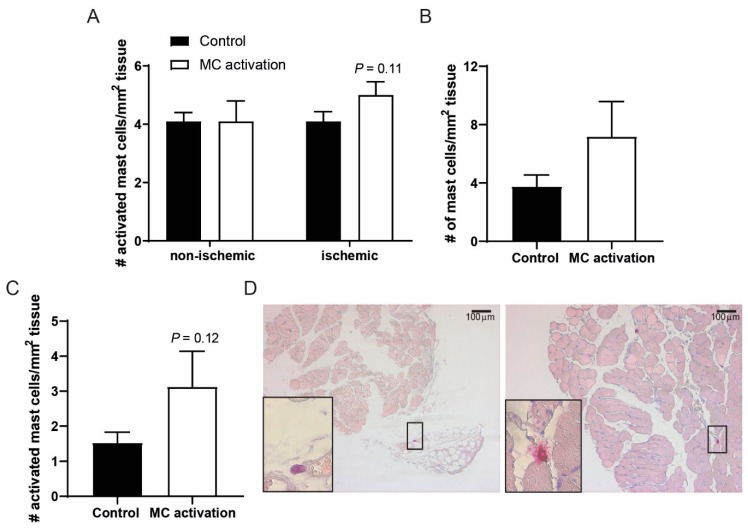
Activated mast cell numbers in muscle tissue of the ischemic hind limb. (**A**) The number of activated mast cells as measured by histology in the ischemic vs. non-ischemic adductor muscles of mice in which mast cells were activated compared to control mice at day 9 after the induction of ischemia. (**B**) Total mast cell numbers per mm^2^ of ischemic soleus muscle tissue. (**C**) Number of activated mast cells per mm^2^ of soleus muscle tissue at day 9 after ligation. (**D**) Representative overview images of the soleus muscles with a resting (control, left) and an activated mast cell (dinitrophenyl hapten, DNP, right) in high-magnification inserts.

We studied to what extent mast cell number and activation status were still affected after the single treatment with DNP of the ischemic hind limb. At nine days after ligation, the number of mast cell observed in the ligated and non-ligated adductor muscles were comparable (Figure 4A). In the ligated adductor muscles, the number of activated mast cells still tended to be increased at nine days after DNP treatment (*p* = 0.11), an effect that was lost at 28 days after ligation. In the soleus muscle of the DNP groups, both total mast cell numbers as well as the number of activated mast cells was still somewhat increased at nine days after ligation as compared to the controls (Figure 4B,C). Representative images of soleus muscle tissue are shown in Figure 4D.

Mast cell activation did not result in muscle hypertrophy, analyzed in both the ischemic and non-ischemic adductor muscles of the control and DNP-group at day 9 and day 28 after femoral artery ligation (data not shown).

### 3.5. Contribution of Arteriogenesis to the Increase in Blood Flow Recovery by Activated Mast Cells

**Figure 5:**
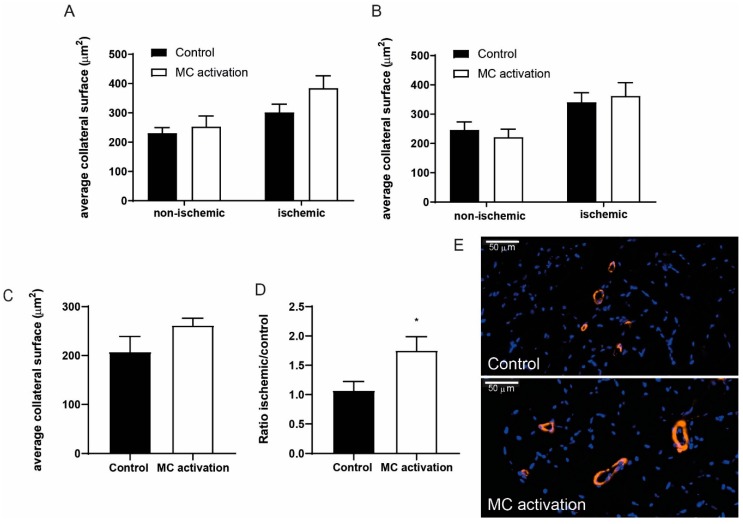
Arteriogenesis upon mast cell activation in the ischemic hind limb. The average collateral surface area in ischemic as compared to non-ischemic adductor muscles of mice in which mast cells were activated compared to controls at (**A**) 28 days after ligation and (**B**) 9 days after ligation. (**C**) The average collateral surface area in the soleus muscle of mice in which mast cells were activated compared to controls. (**D**) The collateral area ratio between ischemic and non-ischemic soleus muscles in mast cell activated compared to control mice at nine days after femoral artery ligation. (**E**). Representative pictures of smooth muscle cell actin positive collaterals in orange and nuclei in blue (DAPI) in both a control muscle and a muscle in which mast cells were activated. * *p* < 0.05.

Since restoration of blood flow recovery is a combination of shear stress induced arteriogenesis and ischemia induced angiogenesis [22], we studied these processes in the adductor muscle upstream of the ligation and in the soleus muscle, the ischemic region downstream of the ligation. Increased shear stress upstream of the ligation leads to arterialization of collaterals in the adductor muscle. At day 28, the number of collaterals did not differ between the ischemic and the non-ischemic adductor muscles in both the control and DNP treated group, nor did the number of collaterals differ between the control and DNP treated group (control: non-ischemic 32.7 ± 1.8, ischemic 28.7 ± 1.8, DNP: non-ischemic 34.6 ± 3.4, ischemic 32.2 ± 1.5). No differences in collateral numbers after ligation is frequently observed in hind limb ischemia studies since the pre-existing collaterals predominantly increase in size upon shear stress. Therefore, we also analyzed the surface area of the collaterals. The area of the collaterals in the ischemic muscles of the DNP treated mice was slightly increased compared to control mice (control: 318.4 ± 12.5 µm² vs. DNP: 381.2 ± 42.5 µm², *p* = 0.205) (Figure 5A). Also, the area of the collaterals in the non-ischemic vs. the ischemic hind limbs expressed as the ratio collateral area was somewhat increased between the control and DNP treated mice, but did not reach significance (control: 1.420±0.1 vs. DNP: 1.611 ± 0.2, *p* = 0.509). 

In the short-term experiment with sacrifice at day 9, the number of collaterals also did not differ between the ischemic and the non-ischemic adductor muscles in both the control and DNP treated group, nor did the number of collaterals between the control and DNP treated group (control: Non-ischemic 27.3 ± 1.8, ischemic 30.7 ± 1.5, DNP: Non-ischemic 29.0 ± 1.8, ischemic 27.3 ± 1.8). Furthermore, no differences could be observed in collateral area in the ligated hind limbs between the control and DNP treated mice at day 9 (control 340.2 ± 29.2 µm² vs. DNP 362.1 ± 45.5 µm², *p* = 0.680) (Figure 5B) nor in the collateral area ratio (control 1.557 ± 0.3 vs. DNP 1.749 ± 0.3, *p* = 0.633).

In the downstream area of the ligation, the distal end of the collaterals in the soleus muscle are also exposed to increased shear stress. Comparable to the adductor muscle, the number of collaterals in the soleus muscle did not differ between the groups with or without ischemia at day 9 (control: Non-ischemic 23.8 ± 2.1, ischemic 26.3 ± 1.7, DNP: Non-ischemic 25.2 ± 2.0, ischemic 23.9 ± 1.9). A non-significant increase in soleus muscle collateral area could be observed in the ligated hind limbs of the DNP treated mice compared to the control mice (control 207.4 ± 31.7 µm² vs. DNP 261.7 ± 14.8 µm², *p* = 0.163) (Figure 5C). Interestingly, the collateral area ratio of the ischemic vs. the non-ischemic soleus of the individual mice was significantly increased in the DNP group vs. the control group (Control: 1.070 ± 0.2 vs. DNP: 1.751 ± 0.2, *p* = 0.026) (Figure 5D), suggesting that local mast cell activation does increase collateral diameter downstream of the ligation.

### 3.6. Effects of Mast Cell Activation on Angiogenesis

**Figure 6:**
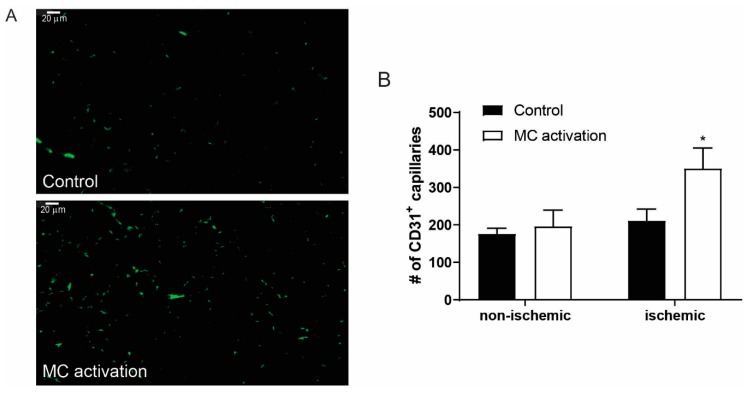
Activated mast cells induce angiogenesis in the ischemic hind limb. (**A**) CD31 staining of the ischemic soleus muscles shows the presence of CD31^+^ capillaries in control mice and in mice in which mast cells were activated. (**B**) Quantification of the number of CD31^+^ capillaries in non-ischemic and ischemic soleus muscle with (white bars) or without local (black bars) mast cell activation, measured at nine days after femoral artery ligation. *p < 0.05.

As a measure for the angiogenic response, CD31 positive capillaries were quantified in the right non-ischemic and the left ischemic soleus muscles. Local mast cell activation resulted in an increased number of angiogenic capillaries in the ischemic soleus muscles (Figure 6A). Quantification revealed a 66% significant increase in the number of capillaries (control: 211 ± 31 vs. DNP: 351 ± 55 µm², *p* = 0.039, Figure 6B), whereas in the control non-ischemic muscles, no significant difference in the number of capillaries were observed (control 176 ± 15 vs. DNP 196 ± 40 µm²). 

### 3.7. Local Mast Cell Activation Induces a Pro-Inflammatory Monocyte Response

**Figure 7:**
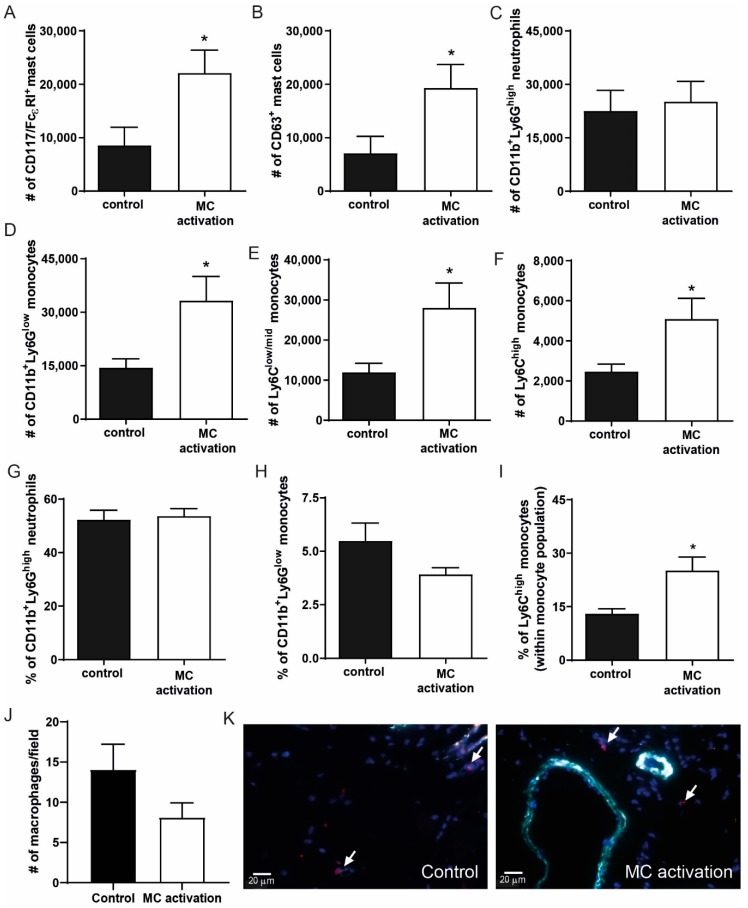
Inflammatory cell analysis in the ischemic hind limb at nine days after ischemia induction. (**A**) Total CD117^+^FcεRI^+^ mast cell numbers and (**B**) the number of CD63^+^ activated mast cells were measured in the inguinal lymph node (iLN) draining from the ischemic hind limb of mice in the DNP-group and the controls using flow cytometry. (**C**) The number of CD11b^+^Ly6C^high^ neutrophils in the iLN. (**D**) The number of CD11b^+^Ly6G^low^ monocytes of which, (**E**) Ly6C^low/mid^ and (**F**) Ly6C^high^ monocytes. (**G**) Percentage of neutrophils, (**H**) percentage of total monocytes, and (**I**) percentage of inflammatory monocytes within the total monocyte population were measured in the circulation of control mice vs. mice in which mast cells were activated. (**J**) The number of macrophages per microscopic field in the ischemic soleus muscles of mice in which mast cells were activated vs. controls at nine days after ligation. (**K**) Representative micrographs of macrophages in red (indicated by arrows) and aSMA^+^ collaterals in cyan and nuclei (DAPI) in blue. * *p* < 0.05.

To determine whether local mast cell activation induced a pro-inflammatory response in the hind limb, we analyzed the inguinal lymph nodes draining from the hind limbs in which mast cells were activated at day 9 after ligation. To do so, we measured the number of CD117^+^FcεR^+^ cells and analyzed CD63 as a common mast cell activation marker. Interestingly, mast cells numbers were highly increased in the DNP group (Figure 7A), similarly to the number of activated mast cells (Figure 7B), which suggests that mast cells have drained from the muscle tissue to the lymph nodes. The number of CD11b^+^Ly6G^high^ neutrophils in the lymph nodes did not differ between the groups (Figure 7C), nor did the amount of T- and B-cells (data not shown). The number of CD11b^+^Ly6G^low^ monocytes in the draining lymph nodes was significantly increased upon mast cell activation (Figure 7D), which was due to an increase in both the Ly6C^low/mid^ and the Ly6C^high^ monocytes (Figure 7E,F). In the circulation, neutrophil and total monocyte numbers were not affected (Figure 7G,H), however within the monocyte population, the relative amount of inflammatory Ly6C^high^ monocytes was significantly increased (Figure 7I). We also measured plasma IL-6 and CCL2 levels as markers of a systemic inflammatory response at 9 days after ligation but were unable to detect any differences between the control mice and those in which mast cells were locally activated (data not shown). To further investigate the local effect of mast cell activation on the pro-inflammatory response in the ischemic tissue, we used a MAC3 staining to quantify the number of macrophages present. Here, in contrast to the number of inflammatory monocytes in the draining lymph nodes, no effect on the macrophage numbers in the ischemic soleus muscles was observed at day 9 (control: 14.1 ± 3.2 vs. DNP 8.1 ± 1.8, *p* = 0.141, Figure 7J,K).

## 4. Discussion

In this study, we aimed to determine the contribution of mast cell activation to angiogenesis in hind limb ischemia. First, we demonstrated the presence of mast cells in human ischemic limb tissue, and both non-activated and activated mast cells were observed. Interestingly, activated mast cells were predominantly present around the capillaries in the ischemic limb, similarly as that described previously in the atherosclerotic plaque, where mast cells were seen to colocalize with microvessels [5]. In addition, mast cells have previously been associated with microvessel density in the brain [23]. Although mast cells have been associated with obesity and diabetes in experimental models of disease [24], the presence of diabetes did not affect mast cell numbers in the human ischemic tissue, which is in line with previous human studies in which intraplaque mast cells numbers did not differ between cardiovascular patients with and without diabetes mellitus [5].

In ischemic mouse hind limbs, the mast cells that colocalized with capillaries were mostly activated early in the revascularization process. Apparently, the ischemic environment in the hind limb causes the mast cells to degranulate. However, the mechanisms via which mast cells are activated upon ischemia remain largely unknown. In ischemia/reperfusion studies, reactive oxygen species, but also alarmins/DAMPS, have been suggested to activate mast cells [24]. The composition of the mast cell secretome completely depends on the specific stimuli and receptors involved, which indicates that the ischemic microenvironment can influence how mast cells are activated and which mediators these cells secrete. In this study, we aimed to determine whether local mast cell activation using a sensitization and challenge protocol in the ischemic hind limb would improve limb perfusion by inducing neovascularization. Similarly as described previously [6], we have established that mast cell activation promotes limb perfusion as indicated by enhanced blood flow to the lower limbs. Previously, it was suggested that neutrophils were the culprit immune effector cells during the neovascularization process in the ischemic hind limb [6]. In our study, we actually show that mast cell activation induced a pro-inflammatory monocyte response, while not affecting neutrophil numbers, either systemically or locally in the draining lymph nodes. The difference in observed immune response between the studies may be explained by the fact that we analyzed these monocyte responses at nine days after the induction of ischemia, whereas neutrophils are usually early responders and act in the first few days after injury. Furthermore, the difference in mast cell activation methods used may have affected the composition of the releasate, resulting in a difference in immune responses in the two studies. The increased immune response in the draining lymph nodes from the ischemic hind limb was not reflected by a difference in macrophage count in the muscle tissue itself at nine days after ligation. Again, this may be due to the timeframe of the study or to local secretion of pro-inflammatory mediators.

To further elucidate the underlying mechanisms that have caused the increased limb perfusion, both angiogenesis and arteriogenesis processes in the ischemic hind limbs were analyzed. In the adductor muscle, we were unable to detect any significant differences in either the number or the surface area of the collaterals, both at 9 and 28 days after femoral artery ligation. In the soleus muscle downstream of the ischemia, however, at day 9 we did observe an increase in the collateral surface area ratio in the mice in which mast cells were activated, suggesting that mast cells increase limb perfusion by expansion of pre-existing collaterals, as arteriogenesis is defined. At the same time point, we observed that the number of CD31 positive capillaries in the soleus muscle was significantly increased upon mast cell activation, and as this did not occur in the non-ischemic muscles, we can conclude that this is a specific local effect of mast cell activation. Together, these effects early in the revascularization process can explain the increase in paw perfusion induced in our model and that both arteriogenesis and angiogenesis in the ischemic muscles are responsible for the increased perfusion. To further elucidate the underlying mechanisms, it would be of interest to study arteriogenesis and angiogenesis processes in mast cell deficient mouse models. However, in our hands, survival of mast cell deficient Kit^W-sh/W-sh^ mice upon hind limb ischemia was too limited to study these processes (unpublished data), and we therefore decided to apply a more therapeutics mast cell activation protocol, which made use of a local delayed type hypersensitivity approach. Previously, we have established that this method induces a specific mast cell activation response [7], however it would also be of interest to study other local mast cell activation approaches to determine which method induces the best neovascularization response without side effects.

As our data imply that mast cell activation improves limb perfusion after an ischemic episode, it is of great interest to determine how we can use these data for therapeutic application. As mentioned earlier, excessive mast cell activation can have detrimental effects on atherosclerotic plaque stability, the lungs, and other tissues in which mast cells reside. Also, an enhanced systemic inflammatory response induced by activated mast cells may be beneficial upon ischemia, but may also negatively affect other sources of inflammation, the so-called Janus phenomenon [13]. Local mast cell activation in the ischemic area may be a more suitable option. Furthermore, the identification of ischemia-specific mast cell activation pathways may provide novel therapeutic strategies to specifically target mast cell activation in the ischemic area. Further research may shed more light on the therapeutic opportunities for intervention.

In conclusion, in this study, we show that mast cells colocalize with capillaries in human ischemic tissue and in ischemic mouse limbs, where mast cells are activated in the early phase after the induction of ischemia. Locally induced mast cell activation in the hind limb leads to an increase in recovery of paw perfusion, which was associated with increased arterio- and angiogenesis, and with an increased inflammatory response, suggesting that mast cells actively contribute to tissue neovascularization.

## References

[1] Kaartinen M., Penttila A., Kovanen P.T. (1994). Accumulation of activated mast cells in the shoulder region of human coronary atheroma, the predilection site of atheromatous rupture. Circulation.

[2] Kovanen P.T., Kaartinen M., Paavonen T. (1995). Infiltrates of activated mast cells at the site of coronary atheromatous erosion or rupture in myocardial infarction. Circulation.

[3] Bot I., Shi G.P., Kovanen P.T. (2015). Mast cells as effectors in atherosclerosis. Arter. Thromb. Vasc. Biol..

[4] Shi G.P., Bot I., Kovanen P.T. (2015). Mast cells in human and experimental cardiometabolic diseases. Nat. Rev. Cardiol..

[5] Willems S., Vink A., Bot I., Quax P.H., de Borst G.J., de Vries J.P., van de Weg S.M., Moll F.L., Kuiper J., Kovanen P.T. (2013). Mast cells in human carotid atherosclerotic plaques are associated with intraplaque microvessel density and the occurrence of future cardiovascular events. Eur. Heart J..

[6] Chillo O., Kleinert E.C., Lautz T., Lasch M., Pagel J.I., Heun Y., Troidl K., Fischer S., Caballero-Martinez A., Mauer A. (2016). Perivascular Mast Cells Govern Shear Stress-Induced Arteriogenesis by Orchestrating Leukocyte Function. Cell Rep..

[7] Bot I., de Jager S.C., Zernecke A., Lindstedt K.A., van Berkel T.J., Weber C., Biessen E.A. (2007). Perivascular mast cells promote atherogenesis and induce plaque destabilization in apolipoprotein E-deficient mice. Circulation.

[8] Sun J., Sukhova G.K., Wolters P.J., Yang M., Kitamoto S., Libby P., MacFarlane L.A., Mallen-St C.J., Shi G.P. (2007). Mast cells promote atherosclerosis by releasing proinflammatory cytokines. Nat. Med..

[9] Indhirajanti S., van Daele P.L.A., Bos S., Mulder M.T., Bot I., Roeters van Lennep J.E. (2018). Systemic mastocytosis associates with cardiovascular events despite lower plasma lipid levels. Atherosclerosis.

[10] Albini A., Bruno A., Noonan D.M., Mortara L. (2018). Contribution to Tumor Angiogenesis From Innate Immune Cells Within the Tumor Microenvironment: Implications for Immunotherapy. Front. Immunol..

[11] van Weel V., Seghers L., de Vries M.R., Kuiper E.J., Schlingemann R.O., Bajema I.M., Lindeman J.H., Delis-van Diemen P.M., van Hinsbergh V.W., van Bockel J.H. (2007). Expression of vascular endothelial growth factor, stromal cell-derived factor-1, and CXCR4 in human limb muscle with acute and chronic ischemia. Arter. Thromb. Vasc. Biol..

[12] Campia U., Gerhard-Herman M., Piazza G., Goldhaber S.Z. (2019). Peripheral Artery Disease: Past, Present, and Future. Am. J. Med..

[13] Epstein S.E., Stabile E., Kinnaird T., Lee C.W., Clavijo L., Burnett M.S. (2004). Janus phenomenon: The interrelated tradeoffs inherent in therapies designed to enhance collateral formation and those designed to inhibit atherogenesis. Circulation.

[14] de Vries M.R., Wezel A., Schepers A., van Santbrink P.J., Woodruff T.M., Niessen H.W., Hamming J.F., Kuiper J., Bot I., Quax P.H. (2013). Complement factor C5a as mast cell activator mediates vascular remodelling in vein graft disease. Cardiovasc. Res..

[15] de Vries M.R., Seghers L., van B.J., Peters H.A., de Jong R.C., Hamming J.F., Toes R.E., van Hinsbergh V.W., Quax P.H. (2013). C57BL/6 NK cell gene complex is crucially involved in vascular remodeling. J. Mol. Cell Cardiol..

[16] Simons K.H., Aref Z., Peters H.A.B., Welten S.P., Nossent A.Y., Jukema J.W., Hamming J.F., Arens R., de Vries M.R., Quax P.H.A. (2018). The role of CD27-CD70-mediated T cell co-stimulation in vasculogenesis, arteriogenesis and angiogenesis. Int. J. Cardiol..

[17] Kraneveld A.D., Buckley T.L., van Heuven-Nolsen D., van S.Y., Koster A.S., Nijkamp F.P. (1995). Delayed-type hypersensitivity-induced increase in vascular permeability in the mouse small intestine: Inhibition by depletion of sensory neuropeptides and NK1 receptor blockade. Br. J. Pharm..

[18] den Dekker W.K., Tempel D., Bot I., Biessen E.A., Joosten L.A., Netea M.G., van der Meer J.W., Cheng C., Duckers H.J. (2012). Mast cells induce vascular smooth muscle cell apoptosis via a toll-like receptor 4 activation pathway. Arterioscler. Thromb. Vasc. Biol..

[19] Aref Z., de Vries M.R., Quax P.H.A. (2019). Variations in Surgical Procedures for Inducing Hind Limb Ischemia in Mice and the Impact of These Variations on Neovascularization Assessment. Int J. Mol. Sci.

[20] Kritikou E., van der Heijden T., Swart M., van Duijn J., Slutter B., Wezel A., Smeets H.J., Maffia P., Kuiper J., Bot I. (2019). Hypercholesterolemia Induces a Mast Cell-CD4(+) T Cell Interaction in Atherosclerosis. J. Immunol..

[21] Wezel A., Lagraauw H.M., van der Velden D., de Jager S.C., Quax P.H., Kuiper J., Bot I. (2015). Mast cells mediate neutrophil recruitment during atherosclerotic plaque progression. Atherosclerosis.

[22] Nowak-Sliwinska P., Alitalo K., Allen E., Anisimov A., Aplin A.C., Auerbach R., Augustin H.G., Bates D.O., van Beijnum J.R., Bender R.H.F. (2018). Consensus guidelines for the use and interpretation of angiogenesis assays. Angiogenesis.

[23] Ollikainen E., Tulamo R., Frosen J., Lehti S., Honkanen P., Hernesniemi J., Niemela M., Kovanen P.T. (2014). Mast cells, neovascularization, and microhemorrhages are associated with saccular intracranial artery aneurysm wall remodeling. J. Neuropathol Exp. Neurol..

[24] He Z., Ma C., Yu T., Song J., Leng J., Gu X., Li J. (2019). Activation mechanisms and multifaceted effects of mast cells in ischemia reperfusion injury. Exp. Cell Res..

